# Do pilocarpine drops help dry mouth in palliative care patients: a protocol for an aggregated series of n-of-1 trials

**DOI:** 10.1186/1472-684X-12-39

**Published:** 2013-10-31

**Authors:** Jane Nikles, Geoffrey K Mitchell, Janet Hardy, Meera Agar, Hugh Senior, Sue-Ann Carmont, Philip J Schluter, Phillip Good, Rohan Vora, David Currow

**Affiliations:** 1 School of Medicine, The University of Queensland, Brisbane, Australia; 2 Department of Palliative and Supportive Care, Mater Health Services, Brisbane, Australia; 3 Department of Palliative Care, Braeside Hospital, Fairfield, Australia; 4 School of Health Sciences, University of Canterbury, Christchurch, New Zealand; 5 School of Nursing and Midwifery, The University of Queensland, Brisbane, Australia; 6 St Vincents’ Private Hospital, Brisbane, Australia; 7 Department of Palliative Care, Gold Coast Health Service District, Gold Coast, Australia; 8 Discipline Palliative and Supportive Services, Bedford Park, Australia

**Keywords:** Pilocarpine, n-of-1 trial, Palliative care, Xerostomia, Advanced cancer

## Abstract

**Background:**

It is estimated that 39,000 Australians die from malignant disease yearly. Of these, 60% to 88% of advanced cancer patients suffer xerostomia, the subjective feeling of mouth dryness. Xerostomia has significant physical, social and psychological consequences which compromise function and quality of life. Pilocarpine is one treatment for xerostomia. Most studies have shown some variation in individual response to pilocarpine, in terms of dose used, and timing and extent of response.

We will determine a population estimate of the efficacy of pilocarpine drops (6 mg) three times daily compared to placebo in relieving dry mouth in palliative care (PC) patients. A secondary aim is to assess individual patients’ response to pilocarpine and provide reports detailing individual response to patients and their treating clinician.

**Methods/Design:**

Aggregated n-of-1 trials (3 cycle, double blind, placebo-controlled crossover trials using standardized measures of effect). Individual trials will identify which patients respond to the medication. To produce a population estimate of a treatment effect, the results of all cycles will be aggregated.

**Discussion:**

Managing dry mouth with treatment supported by the best possible evidence will improve functional status of patients, and improve quality of life for patients and carers. Using n-of-1 trials will accelerate the rate of accumulation of high-grade evidence to support clinical therapies used in PC.

**Trial registration:**

Australia and New Zealand Clinical Trial Registry Number: 12610000840088.

## Background

It is estimated that 39,000 Australians die from malignant disease yearly [1]. Of these, 60 to 88% of advanced cancer patients suffer xerostomia [2], the subjective feeling of mouth dryness. Medications, particularly those with anti-cholinergic side effects such as opioids [3], are the most common cause of xerostomia. Other cases are seen in patients receiving radiotherapy for malignant tumours in the head and neck region as treatment may include salivary glands in their fields causing hypofunction. Patients with reduced salivary flow are at increased risk for dental caries, mucosal breakdown, oral fungal infections, swallowing problems, and diminished or altered taste [4] with secondary symptoms of difficulty in chewing, swallowing and speaking. These symptoms may cause nutritional deficiencies and difficulties in communication and sleeping, leading to overall decline in quality of life [5,6]. Dry mouth symptoms tend to increase towards the end of life [7].

### Pilocarpine

Pilocarpine is a parasympathomimetic agent with predominantly muscarinic activity. Oral pilocarpine formulations are more economical and can be used in lower doses than tablets with reduction in some types of adverse effects [8]. Pilocarpine is very soluble and stable in water solution and the effect lasts for up to 3 hours.

### Efficacy of pilocarpine in reducing xerostomia?

There have been several studies describing symptomatic improvement of dry mouth using pilocarpine in patients with residual salivary function in Sjogren’s syndrome, patients who have received radiotherapy to the head and neck, graft versus host disease, total body irradiation and opioid-induced xerostomia [9-14].

A Cochrane systematic review of pilocarpine for salivary gland dysfunction due to radiotherapy in 2007 [15] suggested that pilocarpine was more effective than placebo, and at least as effective as artificial saliva in those participants that responded (125 (42%) to 151 (51%) from 298 patients). The side effect rate was high (usually the result of generalized parasympathomimetic stimulation) and side effects were the main reason for withdrawal (six to 15% of patients taking 5 mg three times a day). The only study in PC patients, an unblinded single cross-over study, showed that pilocarpine tablets 5 mg tds were more effective than artificial saliva, although they produced more side effects [16].

### N-of-1 trials

**Figure 1:**
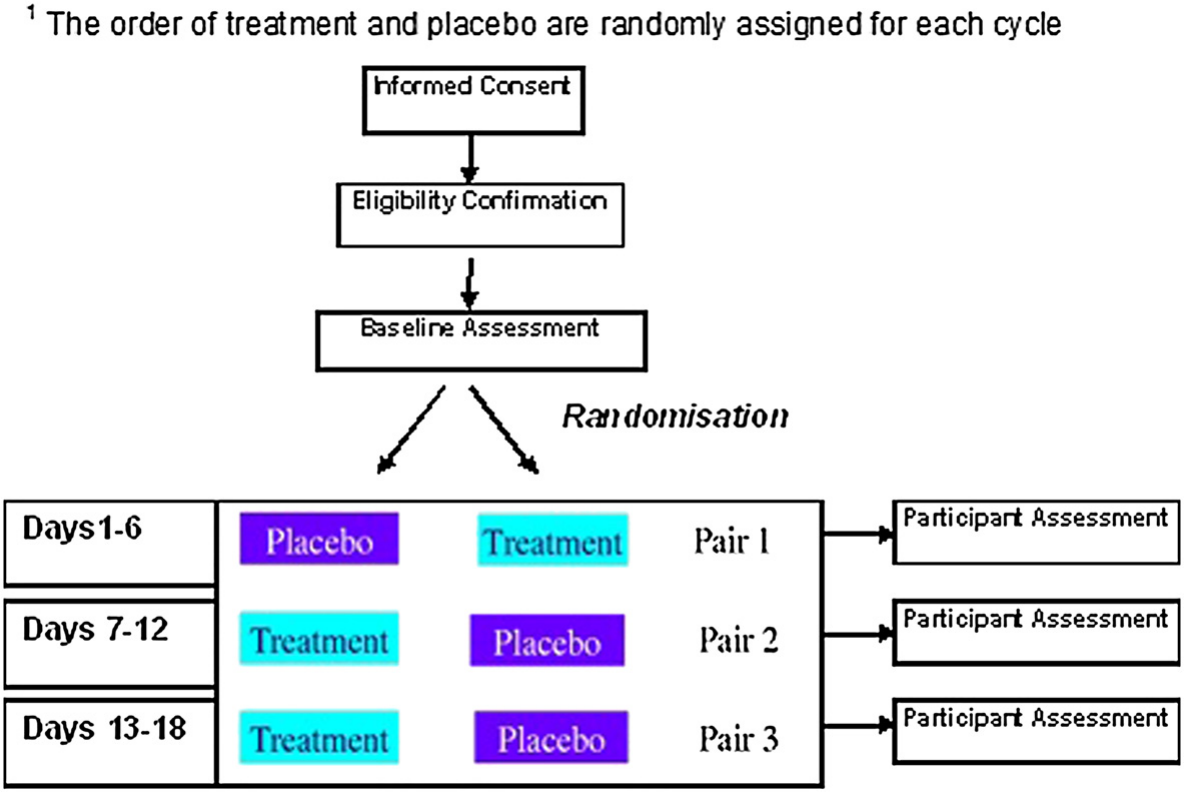
**Example of n-of-1 design schema**^**1**^**.**

The need to improve the evidence base on which PC is based is widely acknowledged [17]. We have previously proposed that n-of-1 trials may provide a mechanism for doing this [18]. N-of-1 trials are multiple-cycle, double blind, placebo-controlled crossover trials using standardized measures of effect (see Figure 1). They provide the strongest evidence possible about the efficacy of a treatment in an individual patient [19]. There are necessary conditions for n-of-1 trials to be conducted, namely: (i) the drug to be tested has a short half-life; (ii) there is no residual impact on the target symptom after excretion; (iii) there is variation in individual response; and (iv) the drug is being used to treat an important and recurrent symptom that has a negative impact on quality of life (QoL). Pilocarpine is a drug ideal for n-of-1 trials: its short half-life allows rapid onset and offset of action; there is variability in response, and it does not change the underlying pathology.

N-of-1 trials are usually used for testing the effectiveness of medicines in individual patients. However, the results of many n-of-1 trials can be combined to determine the effect of a therapy for a population, thus allowing rapid accumulation of strong evidence on treatment effects in patients with advanced life-limiting illness previously very difficult to gather.

## Methods/Design

This study aims to determine a population estimate of the efficacy of pilocarpine drops (6 mg) three times daily compared to placebo in relieving dry mouth in palliative care (PC) patients. A secondary aim is to assess individual patients’ response to pilocarpine and provide reports detailing individual response to patients and their treating clinician.

### Aggregated n-of-1 trial design

This will be an n-of-1 trial with 3 pairs (cycles) of treatment periods comparing active drug to placebo. As pilocarpine has a short half life (0.76 hours for 5 mg tabs), the clinical effect is rapidly evident. Therefore, an appropriate duration of each treatment period is 3 days (thus each treatment pair (or cycle) is 6 days), making a total of 18 days for patients who complete the full trial. The order of drugs in each cycle will be determined by random allocation, blinded to both clinician and patient. Patients who do not complete the full trial will still contribute completed cycles to the final analysis. To produce a population estimate of a treatment effect, the results of all patient cycles will be aggregated [18].

### Setting

Inpatients and outpatients who are eligible will be recruited from 7 hospitals in Queensland and New South Wales, Australia: Ipswich Hospital, Royal Brisbane and Women’s Hospitals, Mater Health Services, St Vincent’s Hospital, Wynnum and Redcliffe Hospitals in Queensland, and in NSW, Calvary Mater Hospital, Newcastle. Ethics approval has been provided by The University of Queensland (UQ) and each site’s institutional ethics committee.

### Participants

a) Inclusion criteria:

1. Patients aged ≥18 years with malignant disease;

1. a clinical diagnosis of chronic dry mouth that has been present for at least 2 weeks with no likelihood of resolution during the trial period

1. a numerical rating scale (NRS) score of ≥3 on a 11-point xerostomia scale;

1. no known allergy or sensitivity to pilocarpine;

1. ability to give fully informed written consent and complete all trial requirements.

b) Exclusion criteria:

1. no plan to change any medication with the potential to cause dry mouth within the trial period. (Patients already on pilocarpine are eligible but must stop this 1 week before trial commencement);

1. no intervention e.g. radiotherapy, chemotherapy, surgery that might alter dry mouth symptoms during the 2 weeks prior to the study period or plans to undergo such therapy during the study period;

1. ocular problems contraindicating the use of parasympathetic agents (eg irido-cyclitis, increased intra-ocular pressure);

1. other comorbidity where there is a risk of worsening co-existing medical problems during the trial period and/or active treatment is contemplated (e.g. severe or uncontrolled asthma or pulmonary disease, uncontrolled hypo-or hypertension, hyperthyroidism, uncontrolled seizures or cardiac arrythmias, (especially bradycardias) and Parkinson's disease);

1. a poor understanding of written or spoken English that would preclude completion of all trial requirements;

1. an active oral infection (e.g. candidiasis, herpetic infections, mucositis, mouth ulcers).

### Screening

Potentially eligible patients [(NRS ≥3/10) or clinical diagnosis of chronic dry mouth] will be identified and screened by research staff. The purpose and requirements of the trial will be fully explained and consent sought.

### Trial medication

a) *Active pilocarpine*

Active pilocarpine hydrochloride 4% (40 mg/ml) in citrus solution; 3 drops orally, three times per day (6 mg per dose) with meals, or identical placebo 3 drops three times daily, will be provided in identical opaque bottles and delivered with a mouth dropper. The dose was chosen based on previous studies [15] and a previous report that pilocarpine (15-30 mg/day) improves symptoms in about 50% of patients, compared 25% of patients taking placebo [20]. Placebo and treatment will have identical taste and color, the strong taste of pilocarpine being masked by citrus. The patients will administer the drops themselves unless assistance is required by a clinical nurse.

Trial packs of bottles will be pre-packed by a pharmacy to allow commencement of the trial as soon as the patient is recruited. The drops are stable for one month. The bottles will be labeled with a randomisation number to keep allocation blinded. Single cycle, 6 day packs will be prepared, with random allocation of the order of the medicines determined by computer, individually numbered and allocated to patients consecutively. The Discipline of General Practice at UQ will run the trial centrally and provide randomised medications and diaries by post to the trial sites for dispensing to patients.

b) *Concomitant therapy*

Regular medications will be continued as required for other conditions. Patients who are prescribed new medicines or increased doses of prior medicines that are likely to affect their xerostomia during a cycle will be withdrawn temporarily from the trial, and data from that cycle discarded. The trial can recommence when effects of the change have been stabilised for at least 1 week.

*Compliance* with the study will be measured by bottle weights and completion rates of the diaries at 18 days and will be encouraged by regular telephone calls from the project officer.

### Primary and secondary endpoints

#### Primary outcome

Symptomatic improvement will be measured by NRS score for average dry mouth (answer to the question – how dry was your mouth on average over the last 24 hours?). A clinically significant response to pilocarpine will be defined as a ≥2 point improvement in xerostomia NRS score compared to placebo. This is in view of previous work, where a 20% (2 cm) improvement or more against the baseline score was considered to be a positive improvement [21,22]. NRS data will be collected on days 2 and 3 of each cycle pair to allow wash-out during day 1.

#### Secondary outcomes

1. Mean xerostomia inventory score [23];

2. Oral health related quality of life;

3. Adverse events (according to NCI Common Terminology for Adverse Events [24]);

4. Dysphagia (difficulty swallowing);

5. Dysgeusia (distortion of taste);

6. Global impression of change.

The trial will be reported according to the Consort statement [25], and analysis will be on an intention-to-treat basis.

### Patient assessment

1. *Outcome Measures*

The schedule of assessments is presented in Table 1. The outcome measures assess different aspects of the trial as follows:

(a) *Response to pilocarpine*:

(i) Numerical rating scale (NRS) for xerostomia. The NRS consists of a range of numbers, with the smaller numbers indicating less dry mouth. An 11-point NRS rates symptom intensity corresponding to an integer number between 0 and 10. People rate their dry mouth by marking a number on a paper that lists the numbers horizontally in ascending order. The NRS has well-established psychometric properties; being valid, reliable, and sensitive to change. It is nonintrusive, easy to administer and score, and suitable for repeated use [26]. Although no studies were found on the psychometric properties of NRS in xerostomia, NRS are commonly used in studies of this condition. Severity of xerostomia will be measured using a 0-10 cm NRS ranging from 0 = no dry mouth to 10 = worst possible dry mouth. At each assessment point, patients will be asked to score their current, worst, best and average dry mouth score over the preceding 24 hours. Dysphagia and dysgeusia (a distortion of the sense of taste) scores will also be recorded in a daily diary using a 0-10mm NRS.

(i) The xerostomia inventory (XI) [23], a valid and reliable scale for measuring xerostomia symptoms, will also be used as a secondary measure.

(a) *Performance status*:

Australian Karnofsky Performance Scale (AKPS) [27]. This scale assesses functional performance and is a validated modification of the gold-standard Karnofsky Performance Scale, altered to apply to both community and hospital patients. It has high test-retest reliability, high predictability of survival time, and sensitivity to change towards the end of life.

(a) *Presence of side-effects*:

Any toxicity will be rated using the National Cancer Institute Common Terminology Criteria for Adverse Events (NCI CTC AE) v3.0 [24]. The trial will be overseen by an independent data safety monitoring committee.

(a) *Quality of life (QOL) indicators:*

(i) EORTC-QLQC15-PAL core items [28], is a general cancer QOL questionnaire suited to a palliative sample as it yields data on overall QOL, physical, emotional and social functioning and other symptoms, has been extensively validated, has reference data available and uses standardised scoring procedures, with evidence concerning interpretation of scores.

(i) Oral health related quality of life will be measured by Oral Health Impact Profile (OHIP) [29], a short 14 item questionnaire that has been validated in a population with xerostomia, although not in cancer or PC.

(a) *Patient Global Impression of Change (PGIC)*:

Is a standardised, validated questionnaire that addresses change in activity limitation, emotions, symptoms and overall quality of life [30].

2. *Timing and content of assessments*

3. *Individual patient reports*

At the end of the trial, the order of medications will be unmasked, and compared with the patient’s observations. Repeated results in the same direction favouring the treatment will be reported in terms of a probability that the observed result is true. The clinical importance of the result will be described by comparing it to a predetermined clinically important change. A clinically significant response to pilocarpine or placebo will be defined as a ≥2 point improvement in xerostomia NRS score compared to baseline, in view of previous work, where a 20% (2 cm) improvement or more against the baseline NRS score was considered to be a positive improvement [21,22]. A report on the patient’s response to pilocarpine will be forwarded to the patient’s doctor so a decision on further treatment can be made.

Palliative care clinicians who recruit participants will most likely also receive the post-treatment report. This potentially may unblind the clinician during the course of the trial in relation to the possible effectiveness of pilocarpine, and particular characteristics of responders. Ideally the clinician recruiting to the study will be a different to the one receiving the results and treating the patient. Collecting data from patients independent of clinicians minimises observer bias.

4. *Withdrawals*

Patients who discontinue the study for whatever reason will be able to resume if their condition can be re-stabilised for at least 1 week. Patients who cannot resume the trial will have their completed cycle results added to the trial dataset for later calculation of the population effect of pilocarpine. If a patient’s withdrawal from the study could be attributed to the intervention, their data will contribute to the analysis under intention to treat principles.

**Table 1:** Schedule of assessments

**Measure**	**Baseline**	**Daily**	**Every 3 days**	**End of 6 day cycle**	**End of trial**
Demographics	x				
Vital Signs	x				
Routine blood test	x				
AKPS	x				x^1^
EORTC-QLQC15-PAL core items	x				x
Adverse Event Reporting^1^	x	x	x	x	x
Xerostomia NRS		x			
Xerostomia inventory		x			
Number of breakthrough salivary substitute uses		x			
Dysphagia		x			
Dysgeusia		x			
Changes in xerostomia related medications		x			
OHIP			x		
PGIC			x		

^1^Telephone assessment conducted by research officer.

### Analysis

#### Sample size calculations

Power calculations were based on the primary outcome variable, the NRS score, and a pooled mean (standard deviation) baseline score across groups of 3.250 (2.975) from Davies et al., 1998 [15] (with correction, 1999). Thomson and Williams’ 2000 [31] article on testing the xerostomia inventory was used to estimate patients’ ICC (intra class correlation). In their table IV, Thomson and Williams give a correlation between standard-question responses that range from 0.20 to 0.76, so we assumed a value of 0.3 (approximately the median). From Bland 2000 [32] [page 204], recognising that r ≈ ICC = *s*_*b*_^2^/ (*s*_*b*_^2^ + *s*_*w*_^2^) where *s*_*b*_^2^ is the between subject variance and *s*_*w*_^2^ is the within subject variance, we can thus determine that *s*_*w*_^2^ ≈ 4.752. Of the 70 palliative care patients randomized by Davies et al. [15], 36 (51%) completed phase I (14 days in length) and 26 (37%) completed phase II (21 days in length after phase I completion). Based on this attrition rate, but recognizing the shorter duration of our study, we expect that approximately 60% of our patients will complete cycle I, 50% will complete cycle II, and 45% will complete cycle III.

If we assume no period effect or treatment × time interaction, then simulations of size N = 10,000 in Stata (Statacorp, College Station, TX) programmed to model the repeated measures design, attrition rates, and *s*_*b*_^2^ and *s*_*w*_^2^ variances above, then n = 70 patients need to be recruited to detect a 2 cm change in NRS scores between treatments with 80% power at the 5% level of significance. With n = 70, we expect 31 patients will complete all 3 cycles, 4 will complete cycles I and II, 7 will complete cycle I, and 28 will fail to complete any cycles. In contrast, a conventional RCT with similar attrition would require 120 patients.

#### Data analysis

Data preparation and descriptive reporting will follow that recommended by the CONSORT statement [25].

For each cycle, data from day 1 will be discarded to allow for a wash-out period, and data from days 2 and 3 data will be analysed. All patients with at least one completed treatment cycle will be included in analyses. An effect size will then be calculated between active medication cycles and placebo, thus providing a population measure of effect commensurate with an RCT.

Both individual and population treatment differences will be estimated using hierarchical Bayesian methods and employing noninformative priors using the methods described in Zucker et al. [33], and Schluter and Ware [34]. The likelihood distributions for each model will be assessed for violations and data transformations undertaken, where necessary. Conventional burn-in periods, model convergence and stability diagnostics, and residual checks will be employed [35]. WinBUGS [35] will be used for the Bayesian analysis.

To describe participants’ overall response, three types of Bayesian results will be presented: (i) the mean of the posterior distribution of the mean difference between placebo and stimulant scores, which gives the best estimate of the overall effect size difference between treatments; (ii) the associated 95% credible region, which give intervals of uncertainty (in this case the 2.5 and 97.5 percentile) of the posterior distributions used in (i); and (iii) the posterior probability of the mean difference that stimulant scores were better than placebo scores, which describes the likelihood that the patients will favour the active treatment in future cycles [34]. A patient will be defined to be a ‘responder’ when these estimated values exceed predefined threshold values [34].

Important confounding variables, such as anti-cholinergic load and cause of xerostomia (eg prior radiotherapy), will in included in adjusted analyses and report treatment effects (or success differences) over the various variable stratifications and combinations, following the method advocated by Zucker and colleagues [33].

## Discussion

Xerostomia is a very frequent and distressing symptom in PC patients with significant physical, social and psychological consequences which compromise the quality of life of patients. Managing dry mouth with treatment supported by the best possible evidence will improve the functional status of patients, and improve quality of life (QOL) for patients and carers. The research question is one of the most important ones in PC. Trial findings will provide evidence to service providers, policy makers and consumers to develop policies and practice around the use of pilocarpine for dry mouth in PC patients.

It is important to assess n-of-1 trial methodology in a range of medications and symptoms and in an array of PC situations. Using n-of-1 trials will help to accelerate the rate of accumulation of high-grade evidence to support clinical therapies used in PC. The method will augment the evidence base for PC clinical therapies, contribute to lessening the uncertainty that surrounds many therapies in advanced cancer, and minimize side effects from treatments without proven benefit. This trial thus forms an important and significant part of the world’s first systematic evaluation of n-of-1 trials in PC.

Moreover, if n-of-1 trial methodology proves to be robust, it will be suitable for many other areas of clinical PC as an alternative method of gathering evidence of similar strength to RCTs, but requiring far fewer subjects to be recruited. N-of-1 trials will accelerate development of the PC evidence base to improve care offered to this very disadvantaged and frail group and make a significant contribution to quality of life for people with terminal illness and their carers.

## Abbreviations

AR: Adverse (drug) reaction; AE: Adverse event; AKPS: Australian karnofsky performance scale; ALT: Alanine aminotransferase; AST: Aspartate aminotransferase; CRF: Case report form; DAP: Data analysis plan; DSMC: Data safety monitoring committee; EORTC-QLQC15-PAL: European Organisation for Research and Treatment of Cancer (EORTC) Quality of Life Group shortened the QLQ-C30 specifically for use in palliative care to a 15-item version entitled the EORTC QLQ-C15-PAL; GCP: Good clinical practice; ICC: Intra-class correlation; IRB: Institutional review board; MPH: Methylphenidate; NCI: National cancer institute; n-of-1: Single patient trials; NRS: Numerical rating scale; OHIP: Oral health impact profile; PaCCSC: Palliative care clinical studies collaborative; PC: Palliative care; QOL: Quality of life; RCT: Randomised controlled trial; SAE: Serious adverse event; SAR: Serious adverse (drug) reaction; SPT: Single patient trials; SUSAR: Suspected unexpected serious adverse reaction; TABS: Tablets; UQ: The University of Queensland; VAS: Visual analogue scale; WHO: World Health Organisation; XI: Xerostomia inventory; XQ: Xerostomia questionnaire.

## Competing interests

The authors declare that they have no competing interests.

## Authors’ contributions

JN, GM, DC and JH conceived the study. All authors contributed to study design. JN drafted the manuscript. PS provided statistical advice and contributed to the writing of the manuscript. All authors approved the final manuscript.

## Pre-publication history

The pre-publication history for this paper can be accessed here:

http://www.biomedcentral.com/1472-684X/12/39/prepub
